# The Weight of Obesity in Immunity from Influenza to COVID-19

**DOI:** 10.3389/fcimb.2021.638852

**Published:** 2021-03-17

**Authors:** Fernanda B. Andrade, Ana Gualberto, Camila Rezende, Nathércia Percegoni, Jacy Gameiro, Eugenio D. Hottz

**Affiliations:** ^1^ Laboratory of Immunothrombosis, Department of Biochemistry, Institute of Biological Sciences, Federal University of Juiz de Fora, Juiz de Fora, Brazil; ^2^ Laboratory of Immunology, Obesity and Infectious Diseases, Department of Parasitology, Microbiology and Immunology, Institute of Biological Sciences, Federal University of Juiz de Fora, Juiz de Fora, Brazil; ^3^ Department of Nutrition, Institute of Biological Sciences, Federal University of Juiz de Fora, Juiz de Fora, Brazil

**Keywords:** obesity, COVID-19, severe influenza, immunity, immunopathology

## Abstract

The severe acute respiratory syndrome coronavirus 2 (SARS-CoV-2) has emerged in December 2019 and rapidly outspread worldwide endangering human health. The coronavirus disease 2019 (COVID-19) manifests itself through a wide spectrum of symptoms that can evolve to severe presentations as pneumonia and several non-respiratory complications. Increased susceptibility to COVID-19 hospitalization and mortality have been linked to associated comorbidities as diabetes, hypertension, cardiovascular diseases and, recently, to obesity. Similarly, individuals living with obesity are at greater risk to develop clinical complications and to have poor prognosis in severe influenza pneumonia. Immune and metabolic dysfunctions associated with the increased susceptibility to influenza infection are linked to obesity-associated low-grade inflammation, compromised immune and endocrine systems, and to high cardiovascular risk. These preexisting conditions may favor virological persistence, amplify immunopathological responses and worsen hemodynamic instability in severe COVID-19 as well. In this review we highlight the main factors and the current state of the art on obesity as risk factor for influenza and COVID-19 hospitalization, severe respiratory manifestations, extrapulmonary complications and even death. Finally, immunoregulatory mechanisms of severe influenza pneumonia in individuals with obesity are addressed as likely factors involved in COVID-19 pathophysiology.

## Introduction

In December 2019, several cases of acute pneumonia of unknown etiology emerged in Wuhan, China. The new pathogen was readily sequenced and phylogenetically related to the previous severe acute respiratory syndrome coronavirus (SARS-CoV). The new coronavirus was therefore termed SARS-CoV-2, the etiological agent of the coronavirus disease 2019 (COVID-19) (Li et al., 2020; Zhou P. et al., 2020). By march 2020 the coronavirus outbreak had emerged to the status of pandemic, significantly challenging human health worldwide (Li et al., 2020; Zhou P. et al., 2020). Clinical manifestations of SARS-CoV-2 infection may vary from asymptomatic infection to mild or severe respiratory syndromes. Approximately 15% of infected individuals develop severe diseases that require hospitalization and ventilation support, whereas 5% of them need admission in intensive care units (ICU) due to complications such as acute respiratory distress syndrome (ARDS), sepsis, thromboembolism, and/or multiorgan failure (Feng et al., 2020).

There are no specific clinical signs to anticipate the progression of mild COVID-19 to severe presentations. However, some pre-established conditions including older age and the presence of comorbidities, such as hypertension, diabetes and obesity were identified as risk factors for severe COVID-19, as previously shown for severe influenza pneumonia as well (Guo T. et al., 2020; Hartshorn, 2020; Shi et al., 2020; Zhou F. et al., 2020). Obesity is a high prevalent disease that has major impact on global health. According to the WHO (2020), overweight and obesity almost tripled from 1975 to 2016, with more than 1.9 billion overweight adults and 650 million ones with obesity. Data from 2016 have shown that almost 40% of the world population were overweight and approximately 13% were obese (Chooi et al., 2018; World Health Organization, 2020). The countries leading the ranking of obesity prevalence in the world are: China, USA, Brazil, India, Russia, Mexico, Germany, UK, Italy and France; and the countries leading the rank of severe obesity are: USA, China, Russia, Brazil, Mexico, Egypt, UK, Germany, Turkey and France (NCD Risk Factor, 2016). In addition, obesity estimates for 2030 antecipates an increse towards 42.1% of the populationin in the African continent, 45.5% in Asia, 36% in Europe, 44.5% in North America, 35.2% in South America and 65.8% in Oceania (Ampofo and Boateng, 2020).

Epidemiological evidences have shown obese COVID-19 patients at increased risk to require hospitalization, ICU admission and to evolve to death (Popkin et al., 2020). This increased susceptibility is positively correlated to the body mass index (BMI) with increasing risk of severity in overweight individuals, stage I obesity and stage II obesity. The BMI is currently used to stratify subjects as overweight (BMI 25 - 30 kg/m²) obesity stage I (BMI 30 - 35 kg/m²), obesity stage II (BMI 35 - 40 kg/m²) and obesity stage III (BMI > 40 kg/m²) (Bhaskaran et al., 2018). Obesity has been highlighted as significant risk factor for COVID-19 severity even at younger ages (Busetto et al., 2020; Malik et al., 2020). Accordingly, overweight and obesity were independent risk factors for severe COVID-19 in patients with type 2 diabetes, a risk that was no longer observed in elderly patients (above 75 years) (Smati et al., 2020). A similar risk has been observed for severe influenza pneumonia among people living with obesity. Epidemiological studies reveal that obesity significantly increases the risk of critical and fatal complications of influenza virus infection (van Kerkhove et al., 2011; Sun et al., 2016). In addition, individuals with obesity have less protection against influenza, and are 2 to 3 times more likely to develop disease than non-obese individuals (Neidich et al., 2017). Obese patients with influenza also take longer to eliminate the virus, which may increase the risk of transmission (Maier et al., 2018). Similar immune processes responsible for the increased susceptibility of obese individuals to influenza, including dysregulated innate immunity, are very likely involved in the increased risk of obese individuals to develop severe COVID-19 (Hartshorn, 2020).

People with obesity are known to present changes at different innate and adaptive immune responses due to chronic low-grade inflammation (Karczewski et al., 2018). These preexisting responses may contribute to increased morbidity and mortality in severe respiratory diseases, since overwhelming cytokine production – known as cytokine storm – is a key pathological phenomenon in severe influenza pneumonia and COVID-19 (Gu et al., 2019; Coperchini et al., 2020; Ye et al., 2020). Metabolic and immunological changes in obesity constitute a favorable environment for the development of cardiovascular disorders, which are strongly associated with COVID-19 and influenza severity and mortality (Mamas et al., 2008; Csige et al., 2018; Fountoulaki et al., 2018; Graupera and Claret, 2018; Guo T. et al., 2020; Nishiga et al., 2020; Shi et al., 2020). Thus, this review aims to address metabolic, immunological and inflammatory disorders in obesity that may contribute to pathophysiological mechanisms involved in severe influenza pneumonia and COVID-19.

## Immune Response in Obesity

Obesity is a non-transmissible chronic disease that has outspread and became highly prevalent in humans worldwide. It is described as a multifactorial and endocrine-metabolic disease that involves interactions among hormonal, genetic and environmental factors triggering adiposity in excess (McCafferty et al., 2020). The adipose tissue is a metabolically active organ, and therefore, featuring obesity just as fat accumulation is an oversimplistic view (Vegiopoulos et al., 2017). The loss of fat storage capacity in dysfunctional white adipose tissue during obesity impairs metabolic and endocrine processes leading to systemic low-grade inflammation in obese individuals. These alterations set the stage for metabolic and cardiovascular complications such as insulin resistance, diabetes, atherosclerosis, hypertension and many cancer types (Karczewski et al., 2018).

### The Dysfunctional Adipose Tissue in Obesity

**Figure 1:**
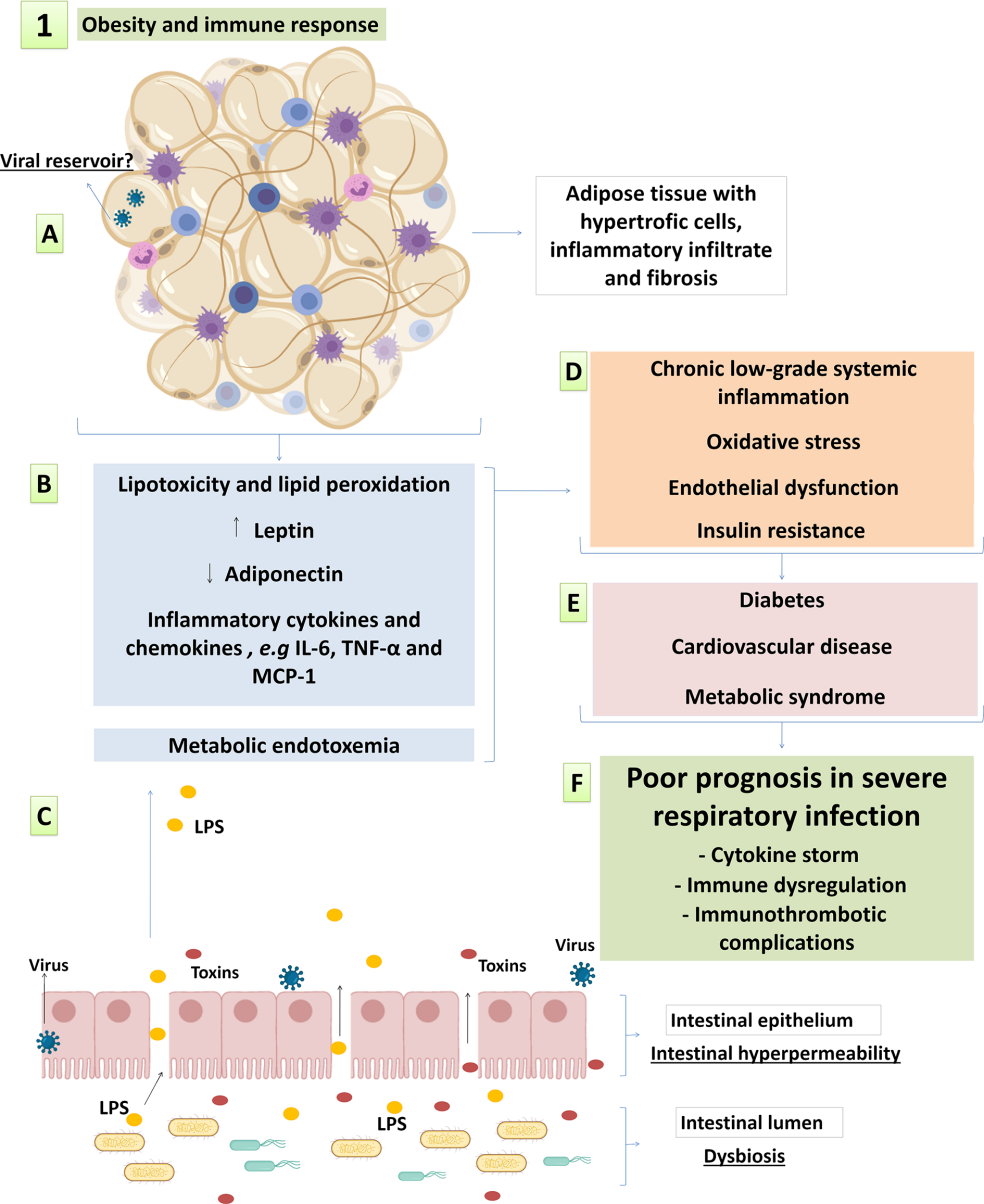
Main factors of obesity-related inflammation and metabolic syndrome possibly involved in pathophysiology of respiratory viral infections. **(A)** changes in adipose tissue architecture with hypertrophic adipocytes, inflammatory infiltrate and fibrosis; **(B)** the loss of fat storage capacity and altered adipokinesecretionin dysfunctional white adipose tissue; and **(C)** metabolic endotoxemia resulting from the intestinal endothelial hyperpermeability also contribute to systemic metabolic and immunological complications; leading to **(D)** systemic low-grade inflammation, metabolic syndrome and increased cardiovascular risk. This syndrome evolves with **(E)** obesity-associated comorbidities and **(F)** increased susceptibility to infectious diseases, including Influenza and COVID-19. See the text for details and references.

Several factors contribute as potential triggers of systemic and adipose tissue inflammation in obesity. First, adipocyte hypertrophy and fat storage capacity loss *per se* is linked to inflammation, as the flow of lipids to non-adipose organs and ectopic fat accumulation lead to lipotoxic effects, tissue inflammation and metabolic dysfunction (**Figures 1A, B**) (Vegiopoulos et al., 2017). In this regard, although the hypertrophy of subcutaneous adipose tissue (SAT) and visceral adipose tissue (VAT) are both positively correlated to metabolic risk factors, VAT expansion is closely associated with adverse metabolic complications (Fox et al., 2007). It has been observed that VAT and perivascular adipose tissue (PVAT) have a twice higher macrophage infiltration than SAT (Kralova Lesna et al., 2016). Macrophages are activated by saturated fatty acids (SFA) through the interaction with pattern recognition receptors (PRRs) of the innate immunity, including Toll Like receptor-4 (TLR-4), TLR-2/1 and TLR-2/6 (Lee et al., 2004; Nguyen et al., 2007). In addition, the pro-inflammatory and pro-oxidant environment of obesity allows oxidation of polyunsaturated fatty acids (PUFAs). Increase lipid peroxidation and circulation of oxidized low-density lipoproteins (oxLDL) are often observed in people living with obesity, and are associated with increased risk for cardiovascular diseases (Njajou et al., 2009; Ayala et al., 2014; Caimi et al., 2019). Lipid peroxidation culminates in the formation of damage-associated molecular patterns (DAMPs) that activate many PRRs present in innate immune cells and other cell types, including in vascular and stromal cells (Miller and Shyy, 2017).

### Inflammatory Infiltrate in Adipose Tissue

Macrophage infiltration is a characteristic of obesity that is positively associated with the BMI and adipocytes size (Weisberg et al., 2003). Macrophages’ infiltration in adipose tissue comes along with the switch from alternatively activated anti-inflammatory (M2) to classically activated pro-inflammatory (M1) macrophages (Lumeng et al., 2007). These two main macrophage phenotypes are the extremes of a broad spectrum of functions that range from tolerogenic to pro-inflammatory properties. Macrophages are polarized to the M1 profile when activated by PAMPS as LPS or proinflammatory cytokines as IFN-γ and TNF-α. The secretion of TNF-α, IL-1β, IL-6 and CCL2 and the increased expression of iNOS are classical M1 profile markers (Murray et al., 2014). M2 polarization is achieved by stimulation with anti-inflammatory cytokines and its hallmarks are the expression of arginase-1 and production of CCL22, CCL17 and IL-10 (Murray et al., 2014). Therefore, M1 polarization in adipose tissue macrophages strongly contributes to the proinflammatory environment in obesity (Lumeng et al., 2007). Alongside macrophages, both B and T cells, from CD4+ and CD8+ subsets, infiltrate the hypertrophic adipose tissue, whereas regulatory T cells reduction is observed, contributing to increased cytokine production, mainly in VAT (Duffaut et al., 2009; Feuerer et al., 2009; Yang et al., 2010; Mclaughlin et al., 2014).

### Adipokines

Besides ectopic fat deposition and lipotoxicity, other adipose-derived mediators are important triggers of inflammation. It is well documented that adipocytes release hormones and cytokines that are collectively classified as adipokines, including leptin and adiponectin, which have pro- and anti-inflammatory effects respectively (**Figure 1B**) (Giralt et al., 2015). Leptin is produced by adipocytes and hyperleptinemia is observed during obesity in parallel to central resistance to its anorexigenic action (Myers et al., 2010). In addition, leptin has several effects on a variety of immune cells, including: 1) increased secretion of proinflammatory cytokines and increased phagocytosis by monocytes, dendritic cells and macrophages (Mancuso et al., 2012; Tsiotra et al., 2012; Moraes-Vieira et al., 2014); 2) increased neutrophil migration (Souza-Almeida et al., 2018); 3) improved cytotoxic activity and IFN-γ secretion in NK cells, but reduced NK cell proliferation and function after long-term exposure (Wrann et al., 2011); and 4) immunometabolic reprogramming of T cells promoting Th1 and Th17 polarization alongside immune tolerance inhibition (Liu et al., 2012; Saucillo et al., 2014; Reis et al., 2015). Adiponectin, on the other hand, is reduced in individuals with obesity. The levels of adiponectin are negatively correlated with visceral fat, insulin resistance, type 2 diabetes and cardiovascular complications (Hoffstedt et al., 2004; Nakamura et al., 2004; Li et al., 2009; Ye and Scherer, 2013). The correlation between low adiponectin levels and obesity-associated comorbidities may be explained by adiponectin’s anti-inflammatory effects, including: 1) macrophage reprogramming from M1 to M2 polarization (Ohashi et al., 2010); 2) TLR-mediated signaling inhibition (Yamaguchi et al., 2005); 3) Suppression of class A scavenger receptor-mediated foam cell formation (Ouchi et al., 2001); and 4) improved endothelial function by increased NO generation and reduced superoxide production (Deng et al., 2010).

Adipocytes also secrete inflammatory cytokines and chemokines as IL-8, IL-6, TNF-α and MCP-1 (**Figure 1B**) (Skurk et al., 2007). Pro-inflammatory cytokines secretion, as well as leptin secretion, are significantly higher in adipocytes presenting greater volume (Skurk et al., 2007). Moreover, it is reported that leptin induces pro-inflammatory cytokine profile in adipocytes, including IL-6 and TNF-α secretion (Palhinha et al., 2019). In addition to increased inflammatory cytokines, the expansion of adipose tissue with hypertrophic adipocytes contributes to impaired vascularization, local hypoxia, fibrosis and cell death (Rausch et al., 2008; Halberg et al., 2009; Spencer et al., 2011). Collectively, these changes in adipose tissue architecture alongside high infiltration of immune cells contribute to chronic low-grade systemic inflammation, metabolic syndrome, oxidative stress and endothelial dysfunction (**Figures 1A, B**) (Weisberg et al., 2003; Curat et al., 2004; Cancello et al., 2005; Vegiopoulos et al., 2017; Graupera and Claret, 2018; Karczewski et al., 2018).

### Intestinal Hyperpermeability and Metabolic Endotoxemia

Intestinal hyperpermeability in individuals with obesity leads to metabolic endotoxemia by increasing the amount of circulating LPS, which is a powerful innate immunity trigger (Damms-Machado et al., 2016). LPS triggers inflammation in TLR4-bearing cells, including macrophages and adipocytes (Song et al., 2006; Medzhitov and Horng, 2009) and may contribute to systemic and adipose tissue inflammation in obese individuals. Metabolic endotoxemia is positively correlated to inflammation, oxidative stress, and macrophage infiltration in the adipose tissue, which were all reduced by antibiotic therapy in high-fat diet (HFD)-fed mice (**Figure 1C**) (Cani et al., 2008a; Cani et al., 2008b). These findings are in line with the “infectobesity” concept, according to which in addition to intestinal microbial agents, viral infections are also associated with adipogenesis and inflammation (Voss and Dhurandhar, 2017; Tian et al., 2019). Even though the cause and effect relationship is not well established, a reciprocal causality may be proposed in which chronic low-grade inflammation in obesity leads to an impaired immunity to pathogens, whereas infections contributes to systemic inflammation and unbalanced lipolysis and adiposity (Voss and Dhurandhar, 2017; Honce and Schultz-Cherry, 2019).

### Metainflammation

It is well established that pro-inflammatory cytokines impair the insulin signaling pathway and consequently lead to insulin resistance (Lackey and Olefsky, 2016). In the absence of insulin orchestration, metabolic impairment advances with increased lipolysis and low glucose uptake. This process leads to hyperglycemia and lipotoxicity, and reciprocally feeds inflammation and oxidative stress (Perry et al., 2015) (**Figure 1D**). This metabolic attachment to the immune response in obesity is called “metainflammation” and is the basis for metabolic and cardiovascular complications, which turns obesity into strong risk factor for chronic diseases such as diabetes, atherosclerosis and hypertension (**Figure 1E**) (Lackey and Olefsky, 2016). It is important highlighting that although inflammatory and metabolic dysfunctions prevail in elderly individuals, they increasingly affect youngsters, who have been more often obese nowadays than in the past. Thus, factors associated with life style, including the ones accounting for weight gain, are causing earlier hyperinflammatory reactions in the general population (Wärnberg et al., 2007). This host homeostasis reprogramming process changes the immune response regulation and the inflammatory environment during infections, including the ones caused by influenza and SARS-CoV-2, over which obesity and metabolic syndrome have major consequences to disease progression, severity and mortality (**Figure 1F**) (Nishiga et al., 2020; Richardson et al., 2020; Wang et al., 2020a; Yang J. et al., 2020).

## Immune Response to Influenza and COVID-19 in Obesity

Epidemiological analyses of COVID-19 have highlighted obesity as a risk factor for severe disease complications. Based on these analysis, the likelihood of individuals with obesity to develop severe COVID-19 syndrome is higher regardless of sex, age and existence of associated comorbidities (Busetto et al., 2020; Malik et al., 2020; Simonnet et al., 2020). Individuals who have obesity are at higher risk of progressing to critical COVID-19 and of requiring admission to intensive care units (ICU) (Sales-Peres et al., 2020), as well as of presenting non-respiratory-related mortality such as shock and acute renal failure (Onder et al., 2020). Recent studies highlight that presenting with overweight is sufficient to increase the risk of hospitalization and even mechanical ventilation during COVID-19, even though the risk is further increased by mild and severe obesity (Lighter et al., 2020; Simonnet et al., 2020; Wang et al., 2020b). In agreement, COVID-19 morbidity and mortality are further increased in obese individuals who present visceral adiposity and ectopic fat deposition (Yang Y. et al., 2020). Regardless the risk of severity, people with overweight and obesity have greater chances of a positive result in SARS-CoV-2 diagnosis, suggesting higher risk of infection and/or symptomatic illness (Eastment et al., 2020; Kim et al., 2020; Wang et al., 2020b; Breland et al., 2021). Similarly, overweight and obesity also increase the risk of having a positive diagnosis for influenza and increased risk of hospitalization for influenza pneumonia, with even higher risk identified among people with severe obesity (Karki et al., 2018).

### Immunopathogenesis

The COVID-19 immunopathological processes are not completely understood, but they may involve similar systemic and airway inflammation processes observed in influenza pneumonia. Severe COVID-19 presentations are associated with imbalance between protective antiviral response and exacerbated pro-inflammatory injury, leading to viral persistence and tissue damage (Felsenstein et al., 2020). The virus can evade innate immunity allowing replication in the initially infected tissues. It is suggested that SARS-CoV-2 infection reduces type I IFN secretion and signaling, pointing to a critical role of IFN-induced anti-viral response in pathogen control (Blanco-Melo et al., 2020; Hadjadj et al., 2020). The lack of virological control may contribute to overwhelmed inflammation and cytokine storm (Tang Y. et al., 2020), which have been associated with COVID-19 and influenza pneumonia main clinical and pathological presentations as ARDS and multiple organ failure (Huang et al., 2020). Along with the cytokine storm, one can observe the disruption of the immune homeostasis with an exacerbated response from the innate immunity, like neutrophils and macrophages, and the impairment of natural killer (NK) and CD8+ T cells, both cytotoxic cells directly involved in viral elimination (Giamarellos-Bourboulis et al., 2020). Thus, patients with severe COVID-19 and influenza pneumonia have irregular immune response that leads to lung injury and increased mortality (Lin et al., 2020; Xu et al., 2020; Zhang et al., 2020a). In this sense, chronic inflammation during obesity may contribute to dysregulated immune homeostasis associated with severe lung injury in SARS-CoV-2 infection, similarly to what is observed in patients with influenza (Luzi and Radaelli, 2020).

**Figure 2:**
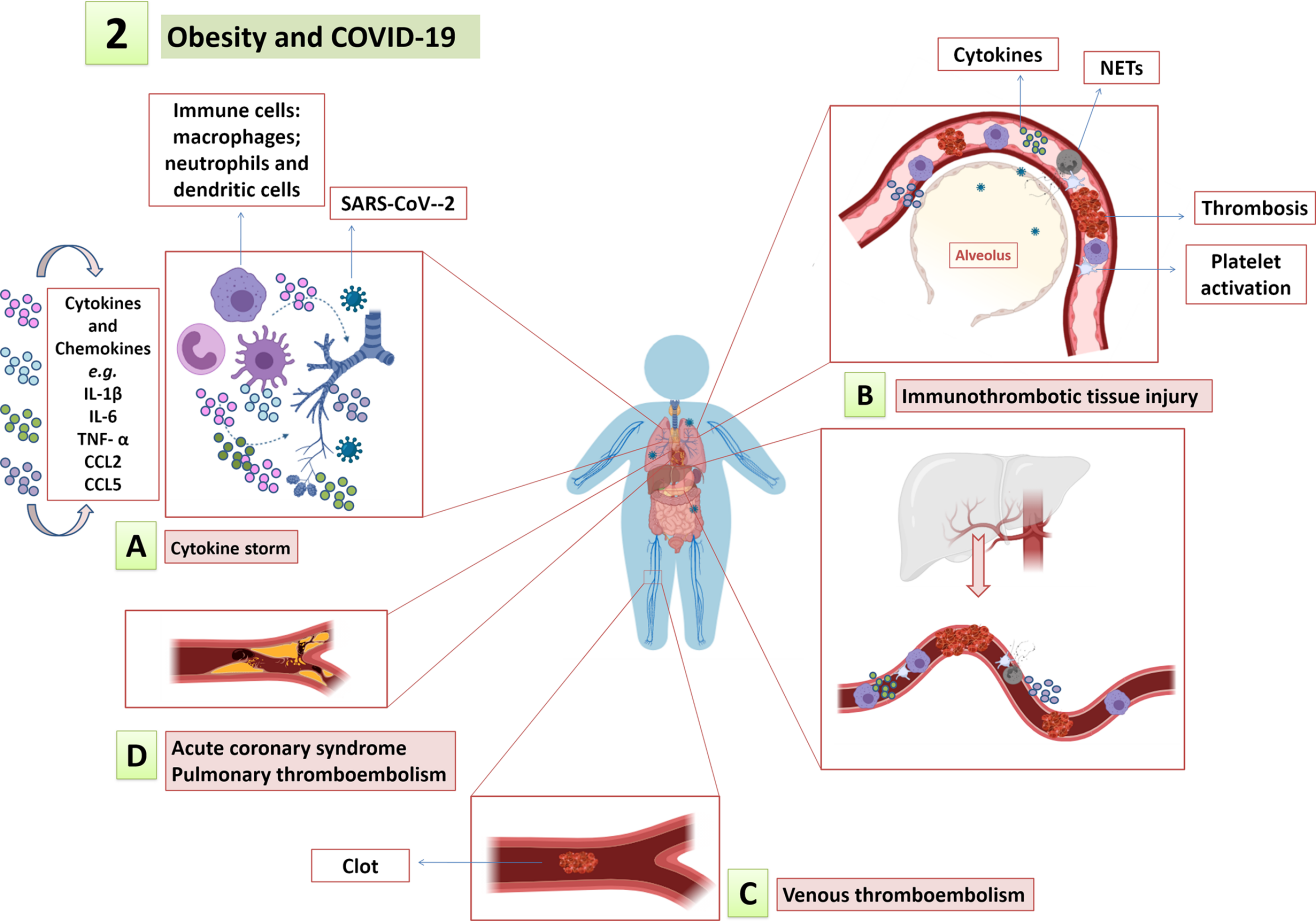
Obesity and COVID-19. The low-grade systemic inflammation, endothelial dysfunction and procoagulant state in obesity amplify and contribute to COVID-19 immunopathology, such as **(A)** cytokine storm; **(B)** immunothrombotic tissue injury; and **(C)** increased risk for thromboembolic events, including **(D)** myocardial infarction, pulmonary thromboembolism, among others.

Obesity-associated chronic low-grade inflammation sets a preexisting release of inflammatory factors and impaired immune system, which increases the susceptibility of individuals with obesity to infections (De Heredia et al., 2012; Hegde and Dhurandhar, 2013). Given the high prevalence of people with obesity among severe COVID-19 patients (Sales-Peres et al., 2020), it is of paramount importance to clarify the immunoregulatory mechanisms of COVID-19 in people with obesity. It is noteworthy that inflammatory cytokines are already increased in people with overweight, including inflammatory cytokines as IL-6, IL-4, MCP-1 and TNF-α. Nevertheless, the levels of the pro-inflammatory cytokines show greater increment in obese individuals and are even higher in severe obesity, since the white adipose tissue itself is a source of these cytokines (Calder et al., 2011; Fan et al., 2019; Pedersen et al., 2019). TNF-α, IL-1 and IL-6 are the main inflammatory cytokines derived from adipose tissue (Skurk et al., 2007) and increased IL-6 levels are predictive of COVID-19 severity and mortality (Ulhaq and Soraya, 2020). Therefore, pro-inflammatory environment prior to SARS-CoV-2 infection as observed in obesity could contribute to amplify the cytokine storm and COVID-19 severity (**Figure 2A**). However, further studies are needed to clarify the role played by IL-6 and other cytokines in individuals with obesity and overweight presenting with SARS-CoV-2 infection.

### The Impact of Metainflammation in COVID-19 and Influenza

Several cytokines and hormones may mediate obesity effects on metabolic reprogramming and impaired effector functions of immune cells. Thus, leptin has several actions in innate and adaptive immunity that can contribute to exacerbate the inflammatory response during respiratory infections. From this viewpoint, leptin is increased in human and murine lungs after severe lung injury due to bacterial sepsis or influenza A (H1N1) pneumonia (Ubags et al., 2015). When it comes to the possible immunoregulatory effects of leptin observed in lung injury models, one can state that leptin plays important roles in recruiting neutrophils to the airways in pneumonia models *in vivo*. This finding corroborates leptin’s likely participation in inflammatory amplification and tissue damage in COVID -19 (Kordonowy et al., 2012; Ubags et al., 2015). Severe COVID-19 patients have increased neutrophil count and activation compared to mild infection, it is also possible observing neutrophil infiltration and extracellular trap (NETs) extrusion in the lungs of critically ill patients (Middleton et al., 2020; Nicolai et al., 2020a). Therefore, we can hypothesize that leptin can also contribute to neutrophils’ infiltration in airways and in other tissues during COVID-19, which also contribute to worsen prognosis in obese individuals. Thus, leptin is an interesting target for future mechanistic research on factors involved in obesity immune dysregulations affecting influenza and COVID-19 pathogenesis.

Chronic inflammation in obesity is followed by immune dysregulation and dysfunctional NK and CD8+ T cells, which also have their function impaired in severe COVID-19 and influenza patients (Sheridan et al., 2012; Zheng et al., 2020). The lipotoxic environment in obesity reduces NK cell counts and impairs NK cells cytotoxic activity, leading to immune paralysis (Michelet et al., 2018). In addition to lipotoxicity, some adipokines as IL-6 and leptin that get higher in obesity also impair the cytotoxic activity of NK cells (Lamas et al., 2013; Cifaldi et al., 2015; Bähr et al., 2017; Bähr et al., 2020). Actually, weight loss in people with obesity increases the secretion of IFN-γ by NK cells (Jahn et al., 2015). In addition, obesity negatively regulates the number of T cell progenitors in the thymus and bone marrow, limits TCR diversification and impairs the metabolic reprogramming of effector CD8+ T cells (Rebeles et al., 2019). Peripheral blood mononuclear cells (PBMC) from people with obesity and overweight show less CD8+ T cell activation and cytotoxic granule release when they are challenged by the influenza virus than cells from eutrophic individuals (Sheridan et al., 2012; Paich et al., 2013). Experimental influenza in HFD-fed mice has been associated with reduced IFN-γ secretion by memory CD8+ T cells in the lungs of mice during secondary influenza infection (Rebeles et al., 2019). Based on the aforementioned processes, obesity-associated inability to produce type I and II interferons during influenza infection allows viral replication and persistence (Honce and Schultz-Cherry, 2019). In order to clarify the association between obesity and influenza virus transmission, some studies have shown that obesity significantly increased the duration of virus shedding by obese adults when compared to eutrophic individuals (Maier et al., 2018), and viral quantification in air samples exhaled by people with influenza showed a positive correlation between obesity and the amount of virus released (Yan et al., 2018). Thus, since immunological changes in obesity during influenza infection increase the risk of severity and potential for transmission, we hypothesize that obesity in individuals with COVID-19 may behave in a similar fashion, which requires new studies. If this hypothesis becomes to be true, it has major implications for social distancing measures, masking and vaccination among people with obesity.

### Is Adipose Tissue a Target for Viruses?

Viruses often take several routes to maximize their infectious potential. SARS-CoV-2 attachment into host cells is known to occur through ACE2 (angiotensin-converting enzyme 2) (Shang et al., 2020). Patients with COVID-19 who present comorbidities such as hypertension and respiratory diseases have higher pulmonary expression of ACE2 (Pinto et al., 2020). Such a fact likely explains how these comorbidities, which are closely related to obesity, contribute to COVID-19 severity (Richardson et al., 2020; Yang X. et al., 2020). Overloading transcription, expression and enzymatic activity of ACE2 have been observed in adipocytes in murine model of diet-induced obesity (Gupte et al., 2008). In addition, a study based on a large cohort of obese patients with COVID-19 showed increased serum ACE2 levels in this population (Emilsson et al., 2020). Individuals with obesity have larger adipose tissue volume and a larger number of cells expressing ACE2, which may favor viral replication. Dipeptidil Peptidase 4 (DPP4) is another receptor involved in SARS-CoV-2 entry found in adipose tissue and in epithelial cells of various organs besides the lungs (Bassendine et al., 2020). Considered an adipokine (Lamers et al., 2011), DPP4 is involved in glucose homeostasis, inflammation and immunity. Experimental obesity in mice showed greater DPP4 release compared to lean. If one takes in consideration DPP4 overloading in obesity (Lamers et al., 2011), increased DPP4 expression may favor SARS-CoV-2 entry in host cells in addition to altering the immunological and metabolic processes involved in COVID- 19 pathogenesis. In addition, studies have shown that SARS-CoV-2 is capable of binding the receptor neuropilin-1 (NRP1), which is abundantly expressed in the respiratory and olfactory epithelium. However, despite satisfactory *in vitro* results, further studies are still needed to clarify the mechanisms of SARS-CoV-2 attachment to NRP1, especially in adipose tissue and neuro-adipose connections (Cantuti-Castelvetri et al., 2020; Daly et al., 2020). The impact of the adipocyte expression of SARS-CoV-2 receptors on the pathogenesis of COVID-19 and the virus likelihood of replicating in adipose tissue are topics that still deserve in-depth investigation.

## Obesity and Increased Cardiovascular Risk in Influenza and COVID-19

As already mentioned, chronic inflammation and oxidative stress in obesity set up a fertile ground for insulin resistance and endothelial injury, increasing the risk of diabetes and cardiovascular complications (Engin, 2017). Accordingly, the high cardiovascular risk (HCR) posed to individuals with obesity could be related to COVID-19 severity. Likewise, HCR can also be involved in increased risk for obesity-associated comorbidities such as hypertension and diabetes (Nishiga et al., 2020; Richardson et al., 2020; Yang J. et al., 2020; Zhou F. et al., 2020). This pre-existing cardiovascular risk may contribute to COVID-19 severity as SARS-CoV-2 infection *per se* can cause complications as acute coronary syndrome and venous and arterial thromboembolism, which are leading causes of mortality (Klok et al., 2020; Nishiga et al., 2020). Accordingly, laboratory analyses point towards significant increase in cardiac injury and coagulation activation markers as predictive of mortality in severe COVID-19 (Tang N. et al., 2020; Zhou F. et al., 2020). The risk of thromboembolic complications as acute myocardial infarction is also higher in influenza virus infection (Kwong et al., 2018; Musher et al., 2019). Nevertheless, alveolar-capillary microvascular thrombosis is almost ten times more frequent in autopsies from COVID-19 fatalities than in those from H1N1 influenza pneumonia (Ackermann et al., 2020). This finding highlights differences in pathophysiological mechanisms linked to the risk of thrombosis between these diseases.

### Vascular Disorders in Influenza and COVID-19

Increased cardiovascular complications of acute influenza pneumonia involve endothelial damage, inflammation and coagulation activation (Fountoulaki et al., 2018; Peretz et al., 2019). Assumingly, cytokine storm in influenza leads to endothelial barrier rupture and vascular hyperpermeability (Wang et al., 2010). Studies conducted with proatherogenic animal models have evidenced that the influenza virus, besides leading to systemic inflammation, also infects and resides in arterial walls and atherosclerotic plaques. Such a process may contribute to mechanisms of cardiovascular complication and thromboembolic events in influenza (Naghavi et al., 2003; Haidari et al., 2010). Extrapolating this process to COVID-19, it is not yet known whether vascular disorders result from a direct effect of the virus on atherosclerotic or normal arteries. However, some findings point towards the presence of the virus in human endothelial cells, and it possibly contributes to the endothelial damage observed in some tissues (lung, kidneys, small bowel, skin) and to multiorgan impairment (Colmenero et al., 2020; Pons et al., 2020; Varga et al., 2020). Endothelial inflammation is a potential trigger for atherosclerosis and represents an initial pathological event of cardiovascular diseases, such as myocardial infarction and other thromboembolic events described in COVID-19 (Gimbrone and García-Cardeña, 2016; Nishiga et al., 2020). Thus, endothelial inflammation could play important roles in cardiovascular disorders caused by COVID-19 and this hypothesis should be strongly considered as pathogenic mechanism.

The mechanisms involved in cardiovascular complications of COVID-19 still need further clarification. Assumingly, the development of cardiovascular and thromboembolic complications due to SARS-CoV-2 infection also involves the direct cytopathic effect of the virus, systemic inflammation and cytokine storm (Sokolowska et al., 2020), which contributes to platelet activation, neutrophils recruitment and endothelial activation/injury, mainly in severe cases (Hottz et al., 2020; Huertas et al., 2020; Middleton et al., 2020). On this regard, increased platelet activation and platelet-monocyte interaction leading to pathogenic tissue factor expression has been associated with hypercoagulability, severity and mortality in severe COVID-19 patients (Hottz et al., 2020). Platelet-neutrophil interactions have been also reported alongside NET extrusion in pulmonary microvascular thrombi from COVID-19 autopsies (Middleton et al., 2020), as well as in other organs (Nicolai et al., 2020a). A recent report have shown higher levels of NET-containing pulmonary vascular occlusive thrombi in COVID-19 than in H1N1 influenza pneumonia (Nicolai et al., 2020b), and this finding suggests differences in the mechanisms of thromboinflammation between influenza and COVID-19.

### Obesity: A Breeding Ground for Cardiovascular Risk During COVID-19 and Influenza

If one takes into consideration that the factors associated with cardiovascular complications in viral infections involve endothelial dysfunction linked to inflammatory and pro-coagulant responses, some pre-existing conditions such as obesity could contribute to cardiovascular complications of the infection. Accordingly, it is well described that chronic inflammation in obesity disrupts endothelial homeostasis (Kwaifa et al., 2020). The endothelial and vascular dysfunction observed in multiple tissues in overweight and obese individuals is closely associated with increased risk for metabolic and cardiovascular disorders (Csige et al., 2018; Graupera and Claret, 2018). Endothelial homeostatic imbalance under these conditions involves reduced availability of vasoprotective and vasodilator molecules, including NO (Förstermann and Sessa, 2012; Xia and Li, 2017), also contributing to inflammatory infiltration (Tabit et al., 2010; Sansbury et al., 2012; Savini et al., 2013; Yao et al., 2017).

Aligned with endothelial dysfunction in obesity, high oxLDL and adipokines levels support the development of atherosclerosis (Berkel et al., 1995; Morange and Alessi, 2013; Chistiakov et al., 2017). Some leptin effects support atherogenesis by increasing oxidative stress, proliferation and matrix remodeling in vascular endothelial and smooth muscle cells, which are linked to plaque vulnerability (Konstantinides et al., 2001; Yamagishi et al., 2001; Li et al., 2005). On the other hand, adiponectin reduction in obesity contributes to increased cardiovascular risk as its protective roles in atherogenesis oppose that of leptin (Okamoto et al., 2002; Yamauchi et al., 2002). Therefore, the association of atherosclerotic process in individuals who have obesity with vascular stress induced by SARS-CoV-2 infection may trigger the rupture of already vulnerable atherosclerotic plaques, leading to arterial thromboembolic events and tissue ischemia. Consistently, platelet activation, which has been associated with COVID-19 severity and mortality (Hottz et al., 2020), is also described in individuals with obesity and is positively correlated with the BMI (Koupenova et al., 2015; Barrachina et al., 2019). Thus, low-grade systemic inflammation, endothelial dysfunction and procoagulant state in obesity could amplify immunothrombotic tissue injury and contribute to thromboembolic events described in COVID-19 (**Figures 2B–D**). A recent study pointed out obesity as strong risk factor for venous thromboembolism in patients with COVID-19 (Hendren et al., 2021). However, BMI effect on cardiovascular outcomes in patients with COVID-19 or influenza remains uncertain and needs to be evaluated in studies with larger and more diverse populations. Besides, experimental animal models could be used to assess the possible cardiovascular outcomes in obese animals with influenza or COVID-19 infection when compared to eutrophic infected animals.

### Metabolic Syndrome, Hyperglycemia and Cardiovascular Risk in COVID-19

Obesity-associated inflammatory and metabolic changes contribute to insulin resistance and increase the risk for diabetes, which, in turns, is a risk factor for COVID-19 severity and mortality (Zhang et al., 2020b; Zhu et al., 2020). Since diabetes has been associated with poor prognosis in COVID-19, the glycemic control of COVID-19 patients has gained great attention. Improved clinical outcomes are observed after better glycemic control of COVID-19 patients with pre-existing type 2 diabetes (Zhu et al., 2020). Although the potential pathogenic link between COVID-19 severity and hyperglycemia are not yet fully clarified, it assumingly involves the HCR associated with inflammation, oxidative stress and endothelial dysfunction under hyperglycemic conditions (Lim et al., 2021). It is well described that hyperglycemia is an independent risk factor for cardiovascular complications (Stratton et al., 2000; Elley et al., 2008). The induction of hyperglycemia acts directly on endothelial dysfunction through pro-inflammatory and pro-oxidative processes described in both diabetes and obesity/overweight (Ceriello et al., 2002; Perkins et al., 2015; Low Wang et al., 2016). The pro-inflammatory environment associated with acute hyperglycemia also impairs the innate immune response, which may harm the fight against SARS-CoV-2 and possibly contribute to COVID-19 poor prognosis (Jafar et al., 2016; Guo W. et al., 2020; Pal and Bhadada, 2020). Some studies suggest that SARS-CoV-2 infection contributes to hyperglycemia and worsens dysglycemia in patients with this pre-existing condition. This finding points to likely bidirectional relationship between COVID-19 and comorbidities as diabetes and obesity/overweight (Apicella et al., 2020; Pal and Bhadada, 2020).

In addition to the physiological vascular consequences of hyperglycemia, a recent study indicates that increased glucose availability contributes for viral replication and leukocyte inflammation in SARS-CoV-2 infection (Codo et al., 2020). Thus, the metabolic reprogramming of monocytes infected by SARS-CoV2 requires the glycolytic metabolism supporting increased inflammatory response, viral replication and pulmonary epithelial cell death (Codo et al., 2020). These data suggest the important participation of glucose metabolism in monocyte-driven exacerbated inflammation, immune dysfunction and lung injury in SARS-CoV-2 infection. Altogether, the abovementioned evidences highlight the well-established contribution of insulin resistance and hyperglycemia to inflammation, oxidative stress and endothelial dysfunction, which breeds the ground for cardiovascular diseases (Petrie et al., 2018). Diabetes involvement in worsening COVID-19 prognosis assumingly involves similar mechanisms as obesity, such as metabolic impairment, pre-existing inflammation, immune dysregulation and cardiovascular disorders (Muniyappa and Gubbi, 2020).

Overall, it is possible suggesting that immune dysregulation, exacerbated inflammation, and high cardiovascular risk could be powerful links between obesity and the severity of influenza and SARS-CoV-2 infection. However, it is essential to better understanding the obesity-related pathophysiological mechanisms exclusively involved in the pathogenesis of severe COVID-19 and the ones shared with influenza virus infection, since it can contribute to improved clinical management and therapeutic strategies applied to individuals with obesity.

## Conclusion

Obesity emerged as important risk factor during viral infections throughout the years, not just because of its persistent proinflammatory condition but also due to its associated metabolic complications and comorbidities, such as diabetes, hypertension, endocrine dysfunctions and immune dysregulation, among others. Altogether, these factors play important roles in worsening viral infections. Better understanding how these conditions contribute to COVID-19 and influenza severity may help decision-making processes at patient screening and management, since early intervention is key to prevent mortality. Besides, it may support the formulation of health policies and hospital beds projections in ICUs and wards. Based on the recent pandemic, it will be essential to stablish stricter surveillance for viral detection and disease morbidity and mortality, by developing therapeutic and clinical strategies for obesity-related mechanisms of pathology in COVID-19 and influenza pneumonia, as well in other infections.

Understanding the mechanisms underlying overwhelming inflammation, cytokine storm and immune dysfunction will be of paramount importance to determine life-style and environmental interventions to mitigate the risk of individuals with obesity to severe respiratory infections. Efforts on future research will solidify the scientific basis and guide health professionals in the clinical management of patients with obesity. While specific therapies and vaccine coverage to control the pandemic are yet to emerge, special care must be provided mainly to the most vulnerable populations, among them elderlies, immunosuppressed patients and people with obesity.

## Author Contributions

FA, AG, and CR wrote the manuscript draft. NP, JG, and EH edited and revised the manuscript. All authors contributed to the article and approved the submitted version.

## Conflict of Interest

The authors declare that the research was conducted in the absence of any commercial or financial relationships that could be construed as a potential conflict of interest.
